# Development of an Online Quality Control System for Injection Molding Process

**DOI:** 10.3390/polym14081607

**Published:** 2022-04-15

**Authors:** Ming-Hong Tsai, Jia-Chen Fan-Jiang, Guan-Yan Liou, Feng-Jung Cheng, Sheng-Jye Hwang, Hsin-Shu Peng, Hsiao-Yeh Chu

**Affiliations:** 1 Department of Mechanical Engineering, National Cheng Kung University, Tainan 700, Taiwan; roy0960@gmail.com (M.-H.T.); adam01172073@gmail.com (J.-C.F.-J.); n16094182@gs.ncku.edu.tw (G.-Y.L.); frankcheng8810@gmail.com (F.-J.C.); 2 Department of Mechanical and Computer-Aided Engineering, Feng Chia University, Taichung 400, Taiwan; hspeng@fcu.edu.tw; 3 Department of Mechanical Engineering, Kun Shan University, Tainan 700, Taiwan; hsiaoyeh@mail.ksu.edu.tw

**Keywords:** injection molding process, pressure curve, switchover position, injection speed, neural network, adaptive process control

## Abstract

This research developed an adaptive control system for injection molding process. The purpose of this control system is to adaptively maintain the consistency of product quality by minimize the mass variation of injection molded parts. The adaptive control system works with the information collected through two sensors installed in the machine only—the injection nozzle pressure sensor and the temperature sensor. In this research, preliminary experiments are purposed to find master pressure curve that relates to product quality. Viscosity index, peak pressure, and timing of the peak pressure are used to characterize the pressure curve. The correlation between product quality and parameters such as switchover position and injection speed were used to produce a training data for back propagation neural network (BPNN) to compute weight and bias which are applied on the adaptive control system. By using this system, the variation of part weight is maintained to be as low as 0.14%.

## 1. Introduction

With the development of industry, the first injection molding machine was invented at the end of the 19th century and developed rapidly in the 20th century. So far, many different types of injection molding machines have been produced and the products become more extensive and more precision. In the injection molding process, there are many factors that will affect the production process. Those factors will lead to the instability of product quality. Consistency is very important to maintain product quality, stable product quality increases production benefits to both producers and customers. In this research, product quality is defined as the variation of the product weight. The factors that will affect the production process include seasonal changes and environmental humidity that directly affect the properties of materials, plastic flow resistance during mold exhaust, cooling or nozzle action, material changes in different batches, human factors, and conservative settings of quality management, etc., which will have a significant impact on product quality [1].

The process of injection molding consists of the following seven stages: plasticization, clamping, filling, packing, cooling, demolding, and ejecting. Among these seven stages, W.-W. Wu et al. [2] proved that the filling stage and the packing stage have a great influence on product weight and the timing between the filling stage and the packing stage is referred to as the switchover point. The switchover point will affect the filling stage, setting the switchover point too early or too late will lead to some problems on product weight. Switchover point can depend on pressure, timing and position. The switchover point that will switch to the pressure holding stage when the screw reaches the specified position during filling is called the position-based switchover point. The most popular method to determine the optimal switchover point is controlled by a screw position. According to the above literature, it can be known that controlling the switchover point can affect the quality of our products and switchover point controlled by a screw position has the best effect. Therefore, this research sets the switchover position as one of the control parameters for adaptive control system.

This research focuses on changes in polymer material properties. J. Wang et al. [3] describes the specific volume on melt pressure and temperature is based on the relationship of P-V-T. Here P is the melt pressure, V is the melt specific volume, and T is the melt temperature. The P-V-T properties of materials affect the quality of injection molding products. J. Wang et al. [4] adopt P-V-T control method and proved this method can be used to accommodate the product weight change due to variation of the melt or mold temperature. Chun-Ying Lin et al. [5] import PID control into pressure and V–P switchover point control to reduce variation of the product weight. In addition to P-V-T control method, Y. Yang et al. [6] present an Injection speed control based on a generalized predictive control, M. Song et al. [7] describe when producing thin-wall products, injection speed plays an important role, and prove Injection speed has a strong effect on the quality of the product. With the literature review above, the proper injection speed is key to molding success. Therefore, this research sets the injection speed as one of the control parameters for adaptive control system.

This research defines the product quality of the injection mode base on the pressure curve. The definition of its quality is to maintain the consistency of product weight, and keep the product appearance from shrinking or the occurrence of short shot. M. Kamiguchi et al. [8] provide a method for monitoring pressure was proposed. C. Collins et al. [9] mentioned the continuous monitoring of cavity pressure changes, the history of cavity pressure is related to the product forming characteristics. Y.-G. Zhou et al. [10] install the pressure sensor in the cavity to obtain accurate data. M.-S. Huang et al. [11] proposed the grey prediction method to determine the switchover position by monitoring cavity pressure in each cycle. Besides mounted pressure sensor in the cavity to measure melt pressure, it can also be mounted at the nozzle. Mounting the pressure sensor at nozzle is more economical than in the cavity. Moreover, S. Orzechowski et al. [12] prove nozzle pressure sensor can also observe the change of the melt pressure at each stage. R. Schiffers et al. [13] used the viscosity index (VI) as an indicator of product quality and to determine the proper switchover position to maintain consistent product quality. Chen et al. [14] pointed out a correlation between changes in viscosity index and the product weight. And develop the control system to reduce variation of the product weight by controlling switchover position. In this research, a pressure sensor mounted on the nozzle was installed to capture the pressure curve of each mold and calculate the peak pressure, the timing of the peak pressure and the viscosity index as the basis of quality.

In intelligent manufacturing control, in order to make the product quality more stable, the network model is used to predict the results. If the results do not meet the expectations, the intelligent control system is used to adjust the control parameters in time to achieve product quality stability. As we all know, injection molding process is a complex nonlinear process, Kitayama, Satoshi, et al. [15] used radial basis function (RBF) network to approximately optimize SAO in the process of plastic injection molding, greatly reducing the simulation time required to obtain the optimal injection speed and pressure profile. Ke, K.C., & Huang, M.S. [16] put the quality indexes related to products into a multilayer perceptron model for learning and prediction and verified the feasibility of the proposed method. Guo, Wei, et al. [17] used the data obtained by the finite element method to train three kinds of BPNN respectively and observed the prediction error and training time of the three methods. Tsai, K.M., & Luo, H.J. [18] combined artificial neural network (ANN) and genetic algorithm (GA) to establish the inverse model of injection molding, and successfully identified the product process parameters with reliable accuracy. LI, Kun, et al. [19] combined BPNN and genetic algorithm (GA) to determine the optimal value of design parameters to minimize warpage.

In order to maintain the consistency of product quality, this research developed an adaptive control system using the BPNN model. The adaptive control system works with the information collected through two sensors installed in the machine only—the injection nozzle pressure sensor and the temperature sensor. The switchover position and injection speed are defined as our control parameters and input of BPNN model. The viscosity index, the peak pressure and the timing of peak pressure are defined as the pressure curve characteristics. The adaptive control system is for online monitoring and real-time parameters control. And the BPNN model is for prediction of the results. This research will discuss whether the system developed in this paper can obtain satisfactory results based on limited information.

## 2. Methods

### 2.1. Injection Molding Process

**Figure 1:**
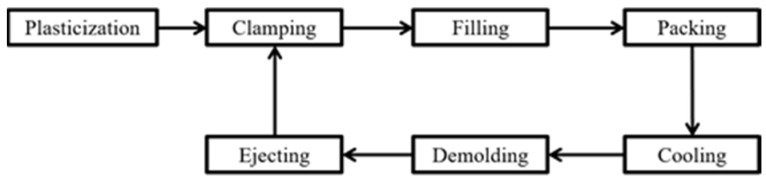
Flow chart of injection molding process.

Figure 1 presents the process of injection molding. The process cycle can be separated into seven parts: plasticization, clamping, filling, packing, cooling, demolding and ejecting.

Plasticization: The material is heated into a molten state through a heated barrel. As the material slowly moved forward by a screw, the plastic is forced into a heated chamber and turn into melt.

Clamping: The clamping mechanism is driven by the oil pressure system to closely fit the fixed side and the movable side of the mold. Therefore, the plastic will not overflow the mold cavity during the molding process.

Filling: The molten plastic is driven out of the nozzle to fill the cavity.

Packing: Before the gate solidifies, it will continue to pressurize to maintain the pressure in the mold cavity to compensate for the volume shrinkage.

Cooling: The molten plastic in the mold cavity will gradually cool and solidify by cooling channel.

Demolding: When the mold is opened, the part shrinks and adheres to the mold.

Ejecting: The product ejected by the ejection system.

### 2.2. P-V-T Relationship

**Figure 2:**
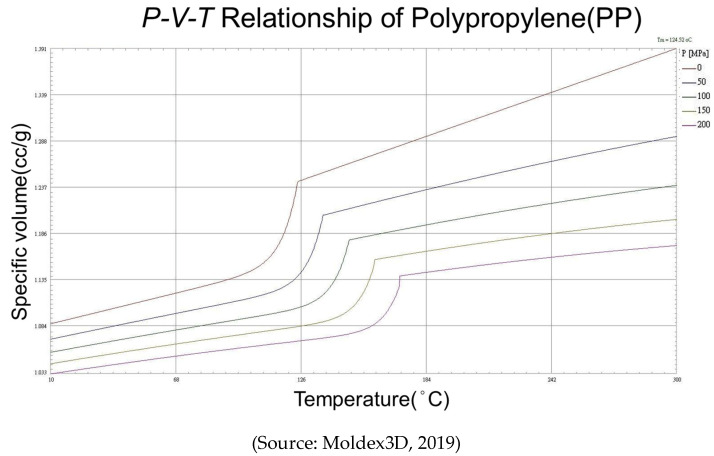
P-V-T Relationship of Polypropylene (PP).

P-V-T (Pressure-Specific Volume-Temperature) polymers properties are important for polymer mechanic. The basic principle is that any two of these state variables determine the value of the third variable. For example, the specific volume can describe the relationship between pressure and temperature. Under constant pressure, when specific volume increase, temperature will also increase too. Figure 2 presents the P-V-T relationship of polypropylene (PP).

### 2.3. Injection Pressure Curve

The method of monitoring the pressure curve is based on the P-V-T relationship. Quality control means maintaining a constant specific volume within a tolerance. Under the stable temperature, if the pressure curve is the same, it can be inferred that the variation of the specific volume is unchanged. Therefore, the weight of the product will be the same, which regarded as the quality indicator.

To describe the material properties during injection molding, this research formulated a viscosity index (VI):

(1) $$VI=\int_{t_{\mathrm{injection}\_\mathrm{start}\;}}^{t_{\mathrm{packing}\_\mathrm{end}}}P_{\mathrm{Melt}\;}\left(t\right)dt$$

**Figure 3:**
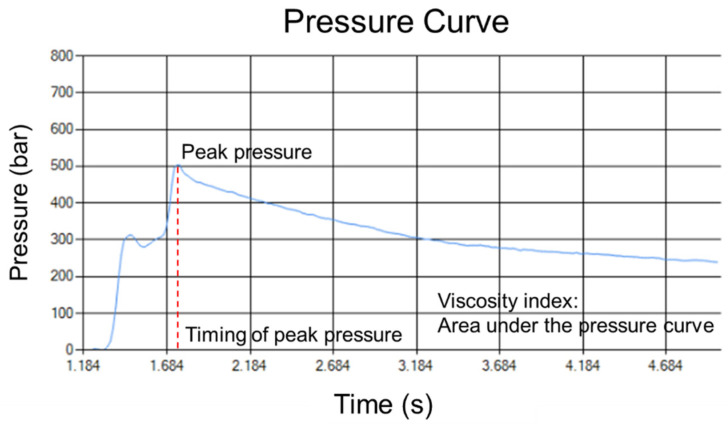
Pressure curve characteristics.

The terms VI, $t_{\mathrm{injection}\text{\_}\mathrm{start}}$, $t_{\mathrm{packing}\text{\_}\mathrm{end}}$ and $P_{Melt}$ represent the viscosity index, injecting start signal, packing end signal, melt pressure, respectively. In order to track the pressure curve, the viscosity index, peak pressure, and timing of the peak pressure are defined as the pressure curve characteristics. Pressure curve characteristics are shown in Figure 3.

### 2.4. Back Propagation Neural Network(BPNN)

#### 2.4.1. BPNN Model

BPNN is an algorithm that adds the backward propagation method to the structure of the feedforward network. Its essence is to transform the input and output problem of the sample set into a non-linear optimization problem. Mainly use the gradient descent method of the chain rule to effectively train the neural network. The main characteristics of the reverse transfer method are iteration and recursion. This method calculates the gradient of the loss function for all the weights in the network and is used to update the weight value to minimize the loss function. The backward pass method is closely related to the Gauss-Newton algorithm. The feedback is used to find the partial derivative. The partial derivative is used to make the gradient descent. The gradient descent is to find the minimum value of the loss function. In summary, the error between the expectation and the output can reduce as much as possible.

**Figure 4:**
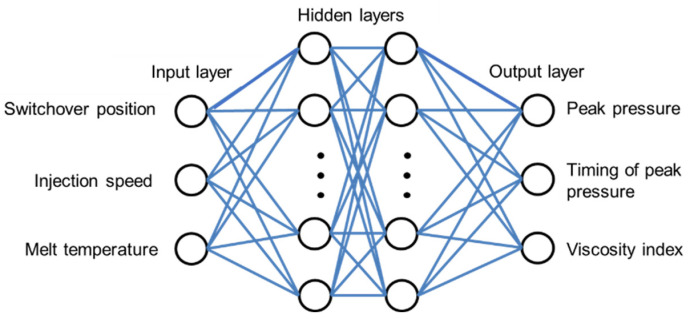
Neural Network Prediction Model.

There are three layers of network structure including input layer, hidden layer and output layer. This research built a 3-60-60-3 BPNN prediction model. The activation function that has been applied in this paper is ReLU that have been mentioned in A.L. Maas et al. [20], and the learning rate (η) is 0.001. The switchover position, injection speed, and melt temperature are inputs. The viscosity index, peak pressure, and timing of peak pressure are outputs. The configuration of the neural network model developed is shown in Figure 4.

The activation function ReLU have no saturation zone, the convergence is fast and the calculation is simple.

(2) $$\mathrm{relu}\left(x\right)=max\left(x,0\right)=\{\begin{array}{c} x,\;\;\;\;x\geq 0\\ 0,\;\;\;\;x<0 \end{array}\in \left[0,+\infty \right)$$

(3) $${\mathrm{relu}}^{\prime }\left(x\right)=\{\begin{array}{c} 1,\;\;\;\;x\geq 0\\ 0,\;\;\;\;x<0 \end{array}\in \left\{0,1\right\}$$

#### 2.4.2. Training Process of BPNN

First, initialize the weights and bias values (random). Second, introduce the current weights into Equations (4) and (5) to calculate the output results. Third, use Equation (6) to calculate the error between the output results and the target results. Finally, using Equations (7) and (8) adjust the weight and repeat the above process until convergence.

(4) $${net}_{j}=f\left(\overset{I}{\underset{i=1}{\sum }}w_{ij}\times net_{i}\right)$$

(5) $$net_{k}=f\left(\overset{J}{\underset{j=1}{\sum }}w_{jk}\times net_{j}\right)$$

(6) $$E=\frac{1}{2}\overset{K}{\underset{k=1}{\sum }}{\left(D_{k}-net_{k}\right)}^{2}$$

(7) $$w_{ij}=w_{ij}+\eta \times \delta_{j}\times net_{i}$$

(8) $$w_{jk}=w_{jk}+\eta \times \delta_{k}\times net_{j}$$

The terms ${net}_{i}$, ${net}_{j}$, ${net}_{k}$, f, w, E, $D_{k}$, η and δ represent the input layer neurons, hidden layer neurons, output layer neurons, activation function, weight, loss function, desired output value, learning rate, output layer variable, respectively.

### 2.5. Adaptive Control System

The controller must be able to adapt to the controlled system with variable or initial uncertain parameters to keep the performance of the whole system unchanged. The basic concept of adaptive control is that the measured signal can be used to predict the control system. The predicted value will change the input signal of the controlled system. Therefore, adaptive control can also be regarded as a control system with real-time parameter prediction.

## 3. Experiment Setups

### 3.1. Experiment Device

#### 3.1.1. Injection Molding Machine

A 60-ton injection molding machine (CLF-60TX, Chuan Lih Fa Co., Ltd., Tainan, Taiwan) was used to fabricate samples by a variety of process parameters. The servo valve (D662-4013, MOOG Inc., New York, NY, USA) used in the oil pressure system with a maximum flow of 250 L/min, a maximum working pressure of 350 kgf/cm^2^ and a response time of 44 ms.

#### 3.1.2. Temperature Sensor

The temperature sensor (Futaba EPSSZL, Futaba Bobbin Co., Ltd., Gifu, Japan) is installed in the mold. The maximum allowable mold temperature is 150 °C and the maximum pressure is 150 MPa. The sensor uses optical fiber for infrared detection. The temperature measurement range is 60~430 °C and the response time can reach 8 ms. The voltage value of each volt of the sensor corresponds to the temperature of 100 °C.

#### 3.1.3. Pressure Sensor

The pressure sensor (Gefran IJ-85179A) is installed at the nozzle of the machine. It can measure the melt pressure in real time. The measured pressure range is 0–3000 bar, and the voltage output range is 0–10 V. The voltage value per volt corresponds to the pressure value of 300 bar. It can withstand temperature up to 350 °C and a response time of 1 ms.

#### 3.1.4. Data Acquisition Module

A data acquisition module (USB-4716, Advantech Co, Ltd., Taipei, Taiwan) with high sampling rate (20 kHz) was used to obtain temperature data and pressure data from the sensor and translate to digital data. The resolution can reach 16-bits, the working voltage is ±10 V, which meets the requirements of pressure and temperature acquisition.

### 3.2. Material

In this research, polypropylene (PP-6331, Lee Chang Yung CHEMICAL CORP., Kaohsiung, Taiwan) was used for experiment with a shrinkage of 1.2%. It is easy to produce serious shrinkage, resulting in the difficulty of weight quality control. It makes our experiment results easy to observe.

### 3.3. Sample and Mold

**Figure 5:**
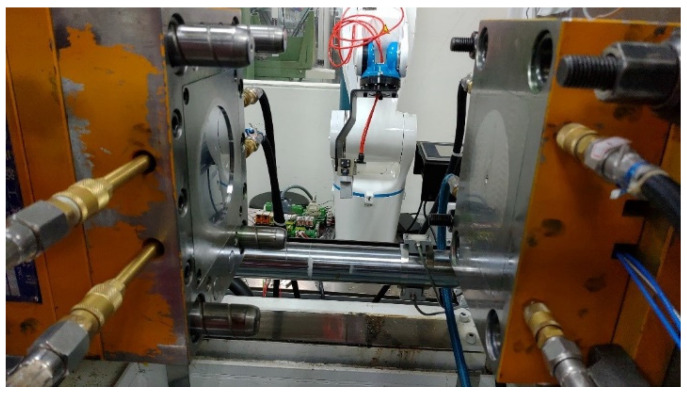
The mold of the thin plastic disk sample.

**Figure 6:**
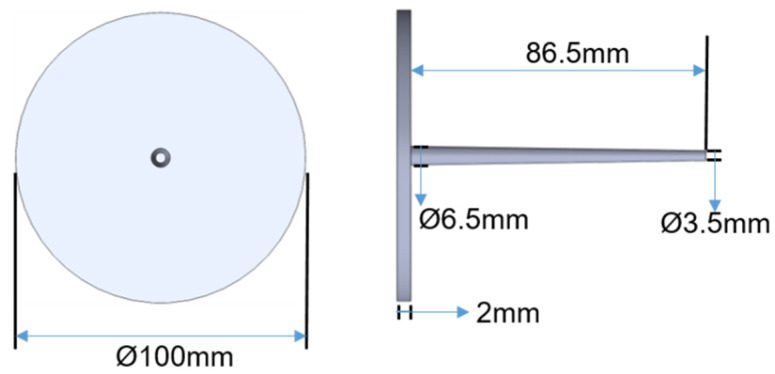
The size of the thin plastic disk sample.

The experiment of this research uses a mold of a thin disc workpiece shown in Figure 5. The thickness of the workpiece is 2 mm and the diameter is 100 mm. The size of the workpiece is shown in Figure 6. The mold is designed as a replaceable mold core. The in-mold temperature sensor is installed near the pouring point in the mold cavity to capture the melt temperature in the mold during the injection process.

### 3.4. Preliminary Experiments

#### 3.4.1. Experiment Parameters

This research designed an experiment to verify the influence of the switchover position and the injection speed on the viscosity index, the peak pressure and the timing of peak pressure. Each run of parameters was subjected to 15 mold experiments. Because the injection molding machine was in an unstable state at the beginning, the first 5 molds are not included in the analysis. Therefore, each run of parameters takes the data of 10 molds and discusses the influence of the variable single factor on the viscosity index, the peak pressure, the timing of peak pressure and weight under the fixed parameters. According to the preliminary experiment results, a neural network prediction model and an adaptive control system are established. The experiment parameters are shown in Table 1.

#### 3.4.2. Single Factor Experiment

According to the P-V-T relationship, as long as the melt temperature maintain stable and the quality characteristics kept unchanged, the specific volume will also remain unchanged. Therefore, the product can be made consistency in product quality. The single factor experiment is conducted to characterize the relationship between the switchover position and the injection speed relative to the viscosity index, the peak pressure, the timing of peak pressure and product weight. The injection molding parameters will take the switchover position and the injection speed as the setting parameters. The experiment parameters are shown in Table 1. The single factor experiment proves that we can maintain product quality by adjusting the switchover position and the injection speed.

#### 3.4.3. BPNN Model Establishment

**Figure 7:**
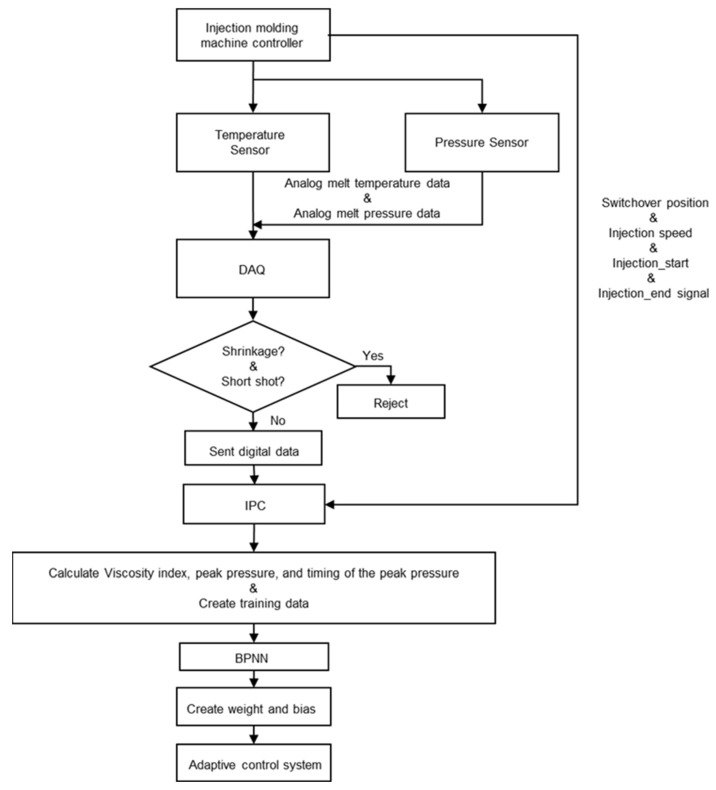
The establishment process of BPNN model.

In order to obtain a neural network model that can predict the results. It is need to train the BPNN model. Since the BPNN model is a supervised learning model, we need to give it the target result for its reference. The target result is obtained by the actual measurement of the machine. Firstly, we conduct the injection experiment by the injection molding machine. The researchers judge whether there is shrinkage or short shot by observing the product. If there is any, it will be rejected. If the product is of good quality, the temperature data and pressure data collected by DAQ from the sensor will be converted into digital data and sent to IPC. IPC calculates and captures viscosity index, peak pressure and the timing of peak pressure by digital data and experiment parameters obtained from DAQ and machine controller. Finally, the generated training data is transmitted to BPNN model to calculate the weight and deviation of adaptive control system. The experiment parameters are shown in Table 1. When the convergence is completed, the model is established. The establishment process of BPNN model is shown in Figure 7.

### 3.5. Flowchart and Parameters of Adaptive Control Strategy

**Figure 8:**
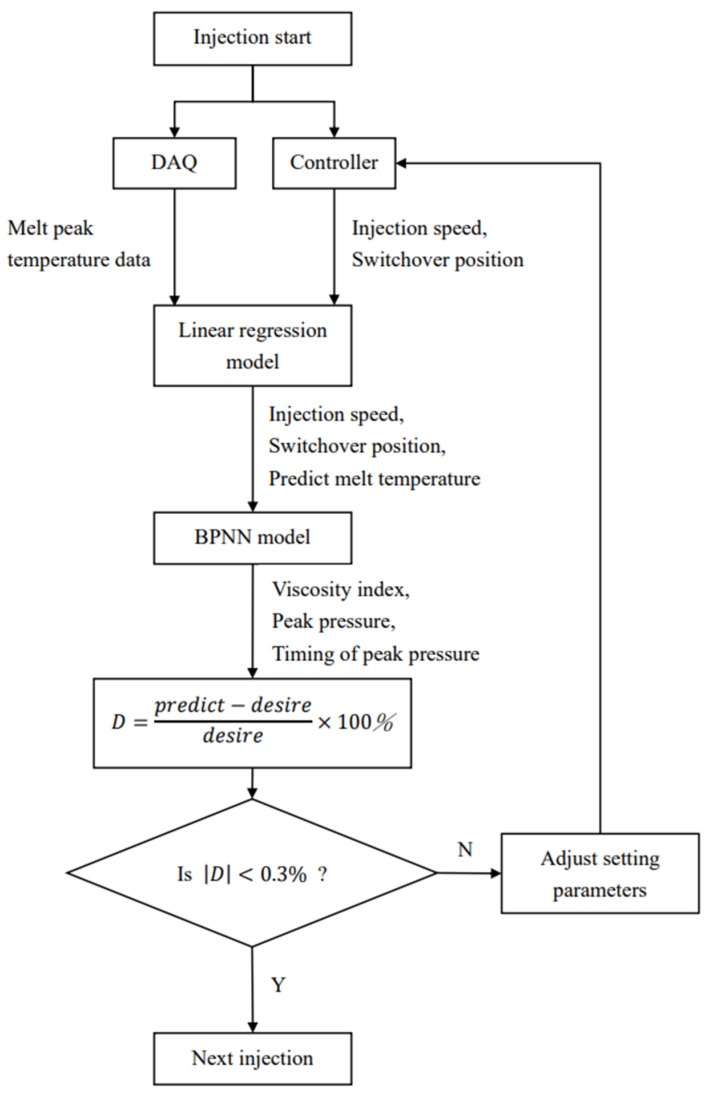
Flowchart of adaptive control strategy.

Firstly, we connect the adaptive control system to the injection machine and start injection. Using DAQ to capture the melt peak temperature data in the mold and use the program to capture the injection parameters of the machine controller (injection speed and switchover position). After capturing those data, we substitute the injection speed, the switchover position and melt peak temperature into the linear regression model in order to predict the temperature of the next cycle. The injection speed, the switchover position and the predicted value of the melt temperature were input into the BPNN model to predict our results, which is, the viscosity index, peak pressure, timing of peak pressure. Next, we compare the predicted results with the target results to determine whether they are within ±3% of the error standard. If it exceeds the defined error range, the switchover position (0.1 mm per time) and the injection speed (1% per time) will be sequentially corrected until the results are within the defined range. Finally, the appropriate injection parameters will return to the machine controller for the next injection. The flowchart of control strategy is shown in Figure 8 and the experiment parameters are shown in Table 2.

## 4. Experiment Results and Discussion

### 4.1. Results of Single Factor Experiment of Switchover Position

**Figure 9:**
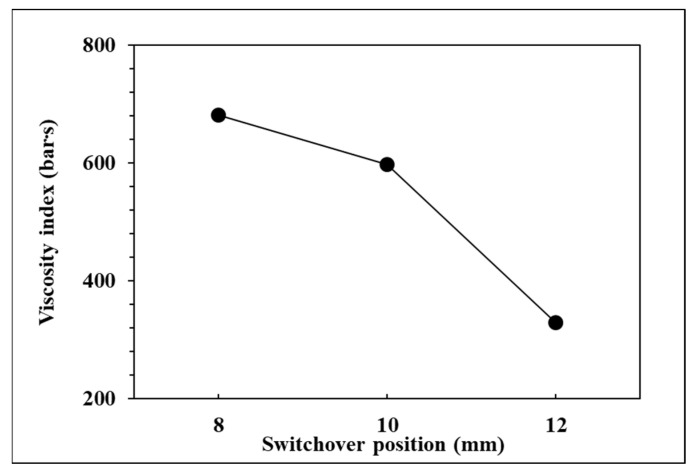
Relationship between viscosity index and switchover position.

**Figure 10:**
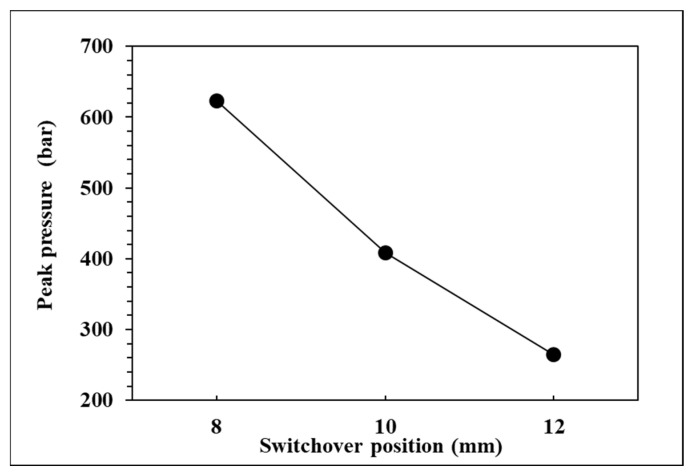
Relationship between peak pressure and switchover position.

**Figure 11:**
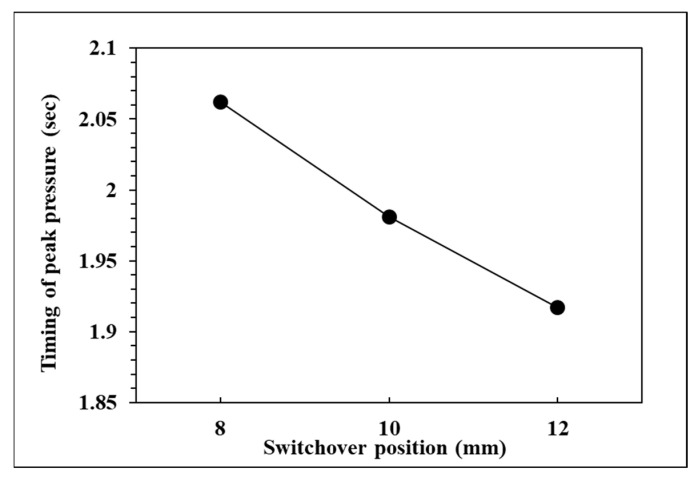
Relationship between timing of peak pressure and switchover position.

**Figure 12:**
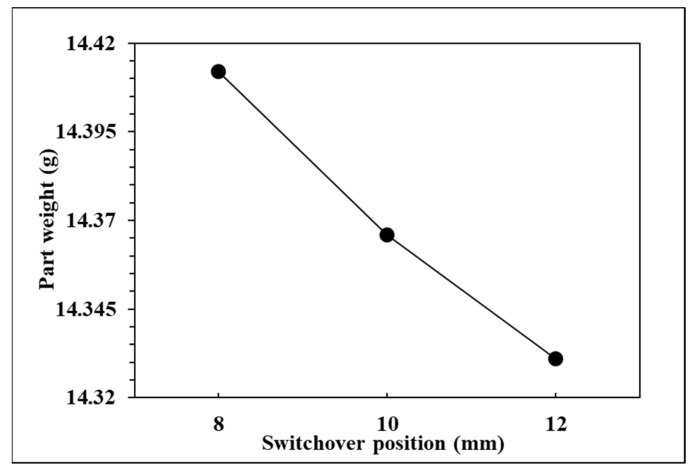
Relationship between part weight and switchover position.

To observe the influence of switchover position on pressure curve characteristics and part weight, the effects of the single factor change of the switchover position were compared by the three groups of experiments, respectively, 12, 10, and 8 mm. The relationships are shown in Figure 9, Figure 10, Figure 11 and Figure 12. If the V/P switchover position is switched early, the timing of peak pressure is shorter than those who switched late. The viscosity index which integrates pressure curve over time is relatively smaller according to the shorter filling time. The earlier the switchover position is switched, the less material will fill into cavity, resulting in the decrease of peak pressure and product weight. Figure 12 displays that the product weight is the heaviest when the switchover position is switched at 8 mm. The experiment results show that the switchover position has the same trend with respect to the viscosity index, peak pressure, timing of peak pressure and the product weight.

### 4.2. Results of Single Factor Experiment of Injection Speed

**Figure 13:**
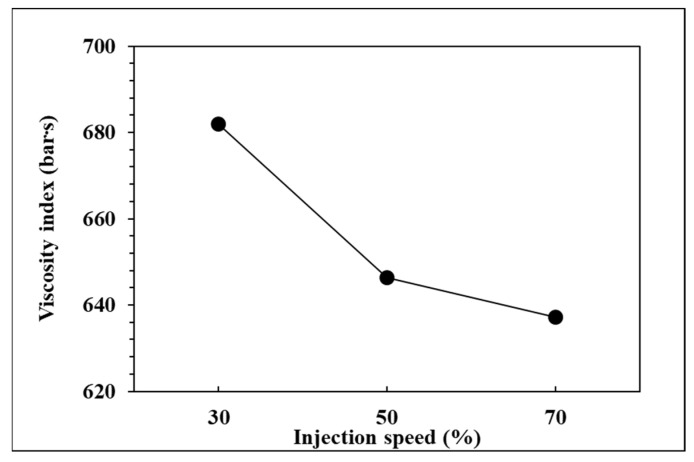
Relationship between viscosity index and injection speed.

**Figure 14:**
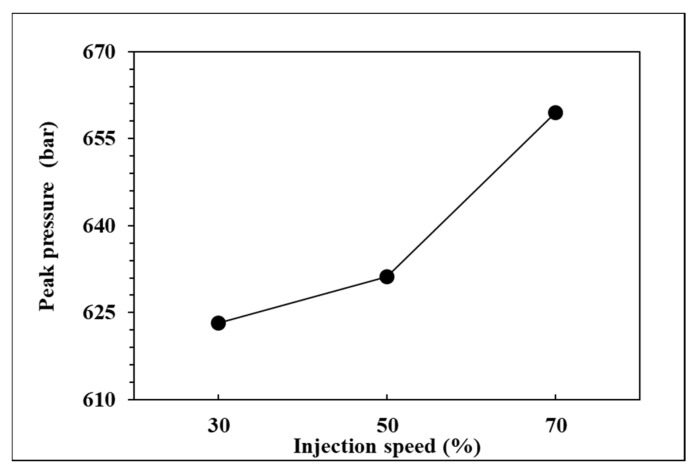
Relationship between peak pressure and injection speed.

**Figure 15:**
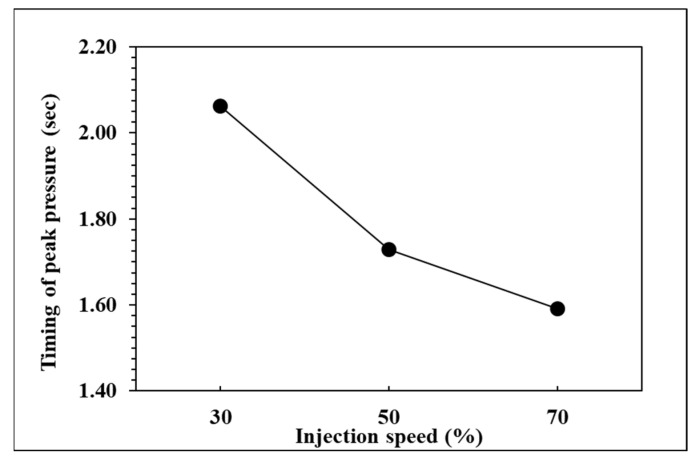
Relationship between timing of peak pressure and injection speed.

**Figure 16:**
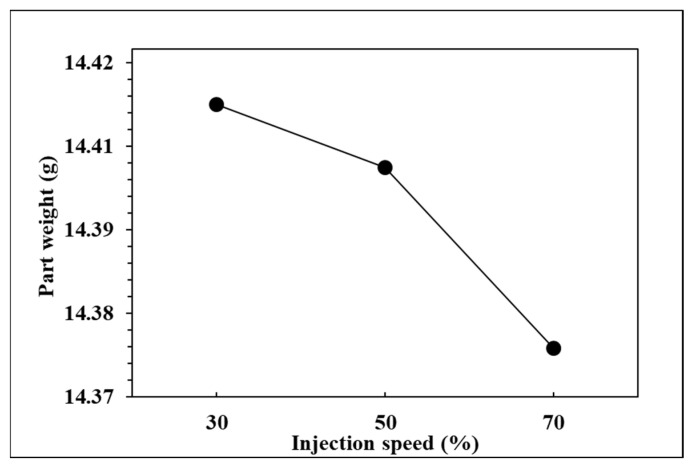
Relationship between part weight and injection speed.

To observe the influence of injection speed on pressure curve characteristics and part weight, the effects of the single factor change of the injection speed were compared by the three groups of experiments, respectively, injection speed in 70%, 50%, and 30%. The relationships are shown in Figure 13, Figure 14, Figure 15 and Figure 16. At the setting of high injection speed, the filling time is relatively shorter than the setting of low injection speed. The viscosity index which integrates pressure curve over time is relatively smaller at the setting of high injection speed according to the filling time. A large injection pressure is required to push the injection speed to the set value of the injection machine, so the peak pressure of the nozzle is relatively bigger at the setting of high injection speed. The faster the injection speed is, the greater the resistance of the melt is, resulting in the decrease of product weight. Figure 16 displays that the product weight is the lightest when the injection speed is 70%. The experiment results show that the injection speed has the same trend with respect to the viscosity index, peak pressure, timing of peak pressure and the product weight.

### 4.3. Results of Neural Network Prediction Model

**Figure 17:**
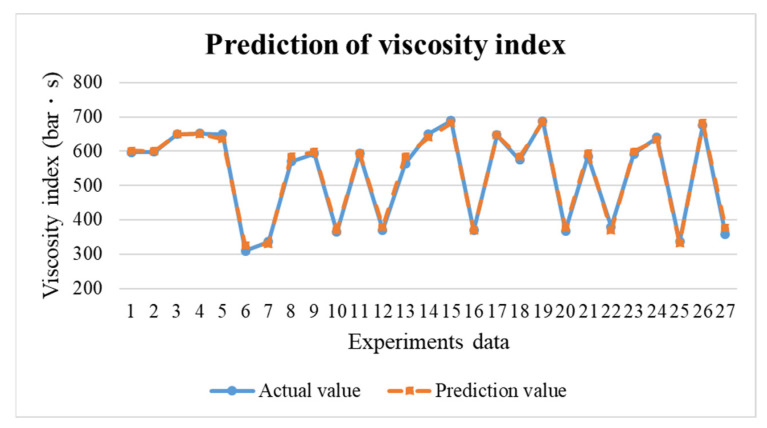
Prediction results of viscosity index.

**Figure 18:**
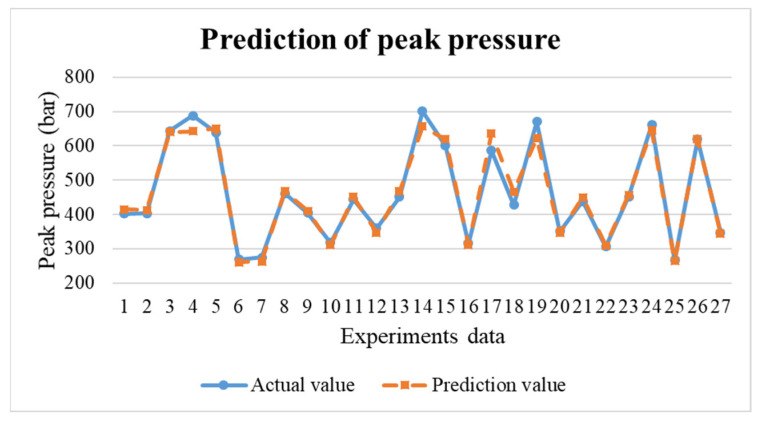
Prediction results of peak pressure.

**Figure 19:**
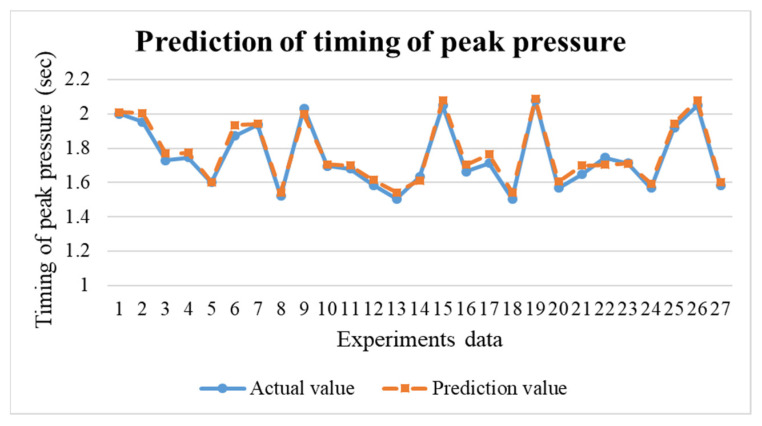
Prediction results of timing of peak pressure.

In the research, the BPNN model is to predict the results for the next mold. If the prediction results are not within the standard range, the process parameters can be adjusted in real time through the adaptive control system to maintain the product weight for each mold. This research uses TensorFlow to build the BPNN model. The neural network were trained by 63 training data obtained in the preliminary experiments. The predictions obtained are shown in Figure 17, Figure 18 and Figure 19. The error values are shown in Table 3. Through the test, the predicted values have positively correlated with the actual values. According to the results of the experiments, an adaptive control system was established through a neural network prediction model.

### 4.4. Experiment Results of Adaptive Control System

**Figure 20:**
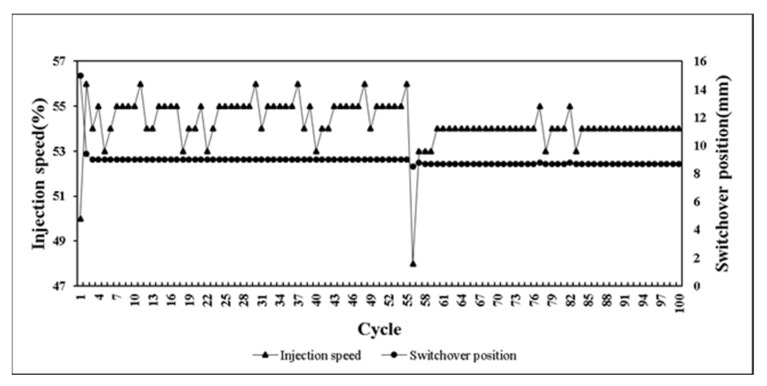
Adaptively adjust parameters results by adaptive control system.

**Figure 21:**
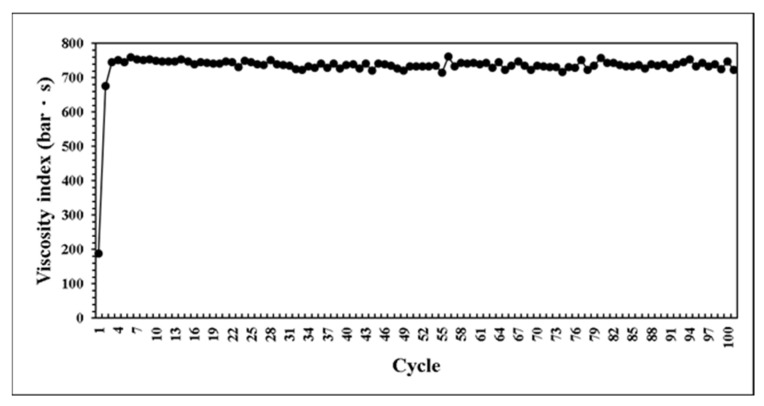
Distribution of viscosity index in experiment.

**Figure 22:**
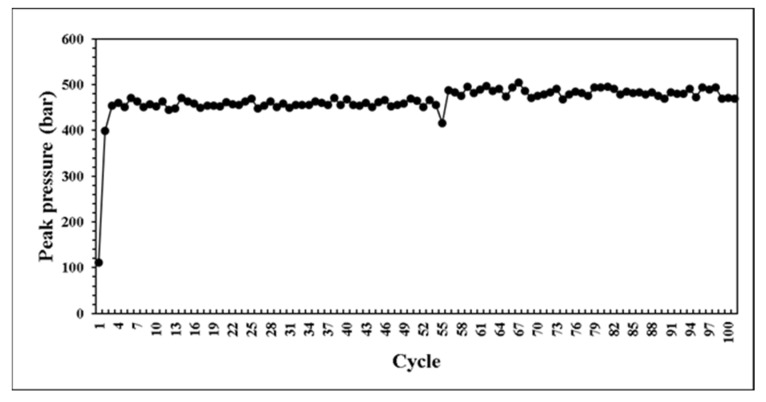
Distribution of peak pressure in experiment.

**Figure 23:**
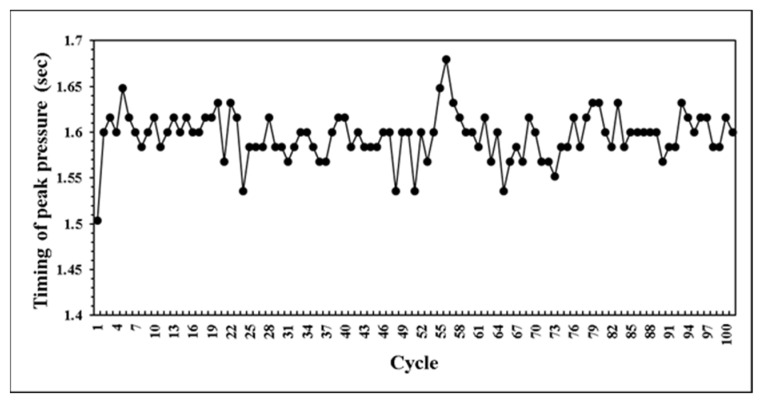
Distribution of timing of peak pressure in experiment.

**Figure 24:**
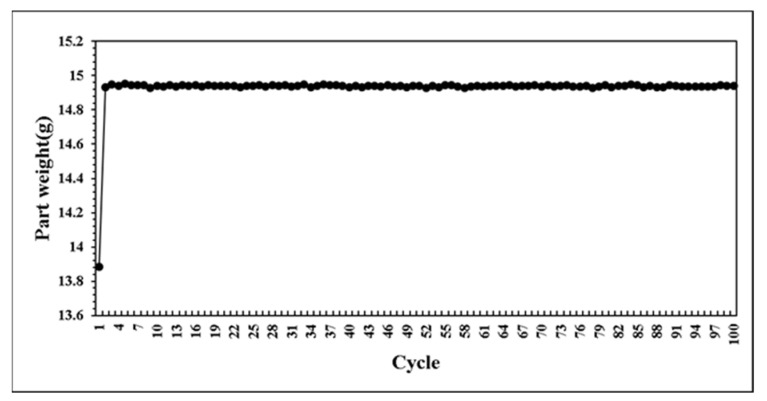
Distribution of part weight in experiment.

This research developed a control system that can self-adjusting switchover position and injection speed according to the variation of melt properties during processing. The system predicts the results of next mold by combining with BPNN model. If the prediction results are not within the standard range, the parameters can be adjusted in real time to maintain the product weight for each mold. At the beginning of the verifying experiment. We first set non appropriate parameters and feedback the proper parameters to machine controller. Figure 20 presents the parameters were adaptively adjusted by the system. Through the experiment results, the variation of pressure curve characteristics are observed. While the system was operating by itself, the variation of pressure curve characteristics is stable which is shown in Figure 21, Figure 22 and Figure 23. Additionally, it was succeeded to maintain the part weight consistency as shown in Figure 24. With further discussion, it can be seen in Figure 21, Figure 22 and Figure 23 that the pressure curve characteristics have not stabilized until the third cycle under the system. Therefore, it is considered that it takes two cycles to stabilize the characteristics. Under the system, the variation of the viscosity index in Figure 21 is controlled within the range of 80 MPa·s. If we focus on the variation after the third cycle, it is controlled within the range of 50 MPa·s. The peak pressure in Figure 22 is more unstable than other characteristics. It is presumed that the injection speed and the switchover position will affect each other while adjusting. Therefore, it is not easy to accurately stabilize the peak pressure. The timing of peak pressure is stable and is controlled within the range of 0.15 s. The weight of the product is stable and has no downward trend. From the above experiment results, the adaptive control system achieves a good performance.

### 4.5. Performance Comparison between with and without System

**Figure 25:**
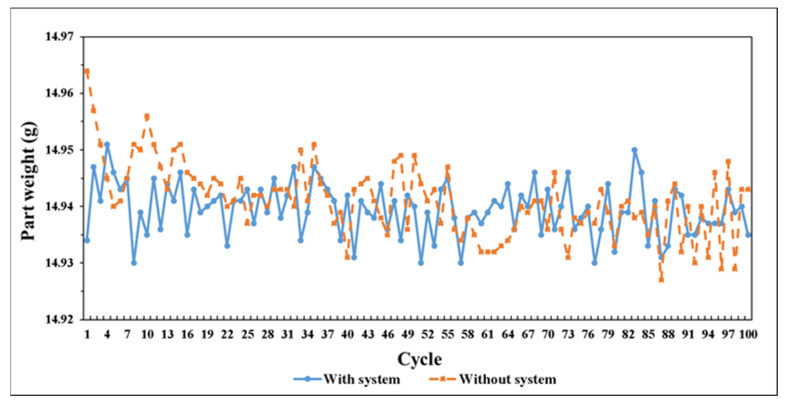
Performance comparison between using system and no system.

To verify performance of adaptive control system, compare the 100-cycle-running results with and without the control system in Figure 25. The respective analysis results are shown in Table 4 and Table 5. These results imply that the system can significantly stabilize the injection molding and maintain the part weight consistency. The variation of part weight decreased from 0.25% to 0.14%.

## 5. Conclusions

This research uses preliminary experiment to prove the correlation between pressure curve and product weight. The result is used to establish an adaptive control system which can adaptively maintain the consistency of product weight by making the pressure curve consistent during processing. By the verifying experiment, compare performance between using system and without system, the variation of part weight decreased from 0.25% to 0.14% under the system control. The result show that injection molding machine with adaptive control system had better performance than without system and achieve the purpose of product weight quality consistency.

## Figures and Tables

**Table 1:** Parameters of preliminary experiment.

Injection Pressure (bar)	Clamping Force (ton)	Packing Pressure (bar)	Packing Time (s)
170	30	15	2
**Run**	**Injection Speed (%)**	**Switchover Position (mm)**	**Melt Temperature (°C)**
1	70	12	210
2	50		
3	30		
4	70	10	210
5	50		
6	30		
7	70	8	210
8	50		
9	30		

**Table 2:** Parameters of adaptive control system.

Injection Pressure (bar)	Clamping Force (ton)	Packing Pressure (bar)	Packing Time (s)
170	30	15	2
**Melt Temperature** **(°C)**	**Injection Speed (%)**	**Switchover Position** **(mm)**	**Cooling Time (s)**
210	50	15	2

**Table 3:** Error of prediction results.

	Viscosity Index (bar·s)	Peak Pressure (bar)	Timing of Peak Pressure (s)
**Average error (%)**	1.64	1.6	3.01
**Maximum error (%)**	3.33	5.12	8.97

**Table 4:** Analysis results of pressure curve characteristics and part weight without adaptive control system.

	Viscosity Index (bar·s)	Peak Pressure (bar)	Timing of Peak Pressure (s)	Part Weight (g)
**Average**	737.562	447.678	1.591	14.9411
**Standard deviation**	14.7182	10.473	0.041	0.0064
**Variation**	13.43%	20.31%	12.07%	0.25%

**Table 5:** Analysis results of pressure curve characteristics and part weight with adaptive control system.

	Viscosity Index (bar·s)	Peak Pressure (bar)	Timing of Peak Pressure (s)	Part Weight (g)
**Average**	737.881	468.735	1.597	14.9395
**Standard deviation**	11.361	17.064	0.0242	0.00461
**Variation**	11.60%	22.31%	9.02%	0.14%

## Data Availability

The data presented in this study are available on request from the corresponding author.
